# A catechol biosensor based on electrospun carbon nanofibers

**DOI:** 10.3762/bjnano.5.39

**Published:** 2014-03-24

**Authors:** Dawei Li, Zengyuan Pang, Xiaodong Chen, Lei Luo, Yibing Cai, Qufu Wei

**Affiliations:** 1 Key Laboratory of Eco-Textiles of Ministry of Education, Jiangnan University, Wuxi 214122, P. R. China

**Keywords:** biosensor, carbon nanofibers, catechol, electrospinning, laccase

## Abstract

Carbon nanofibers (CNFs) were prepared by combining electrospinning with a high-temperature carbonization technique. And a polyphenol biosensor was fabricated by blending the obtained CNFs with laccase and Nafion. Raman spectroscopy, Fourier transform infrared spectroscopy (FTIR) and field emission scanning electron microscope (FE-SEM) were, respectively, employed to investigate the structures and morphologies of the CNFs and of the mixtures. Cyclic voltammetry and chronoamperometry were employed to study the electrocatalysis of the catechol biosensor. The results indicated that the sensitivity of the biosensor was 41 µA·mM^−1^, the detection limit was 0.63 µM, the linear range was 1–1310 µM and the response time was within 2 seconds, which excelled most other laccase-based biosensor reported. Furthermore, the biosensor showed good repeatability, reproducibility, stability and tolerance to interferences. This novel biosensor also demonstrated its promising application in detecting catechol in real water samples.

## Introduction

Nowadays, carbon nanomaterials attract a great deal of attention due to their high surface area, excellent electronic conduction and biocompatibility. Among these, mesoporous carbon [1–8], activated or porous carbon nanofibers [9–19] have been widely studied. Notably, the carbon nanofibers (CNFs) possess a history of more than a century, the carbon filaments discovered in 1889 may be the earliest CNFs [20]. After more than a century of development, various methods used for CNFs preparation are established, such as arc-discharge [21], laser ablation [22], chemical vapor deposition (CVD) methods [23]. Electrospinning, which is known as a facile and convenient process, can produce nanofibers or microfibers with different diameters while using a variety of polymers. The carbonization of electrospun polyacrylonitrile nanofibers can be employed to fabricate CNFs [24]. Lin et al. reported that an electrospun-CNF-modified carbon-paste electrode (CNF–CPE) could be used for the mediatorless detection of NADH [25]. Electrodes modified with Pd/CNFs showed excellent electrocatalytic activities towards dopamine (DA), uric acid (UA) and ascorbic acid (AA) [26]. NiCF-paste (NiCFP) electrodes displayed excellent electrocatalytic capacity for the oxidation of glucose [27]. These works indicate that electrospun CNFs (ECNFs) harbor excellent electrocatalytic properties. However, it is rarely reported that ECNFs were utilized directly in the design of enzyme-based biosensors.

Phenolic compounds, which widely occur in processes of agriculture and industry, often cause severe health problems in human beings and animals [28]. So it is important to develop fast and effective methods to detect phenolic compounds. Laccase (benzendiol:oxygen oxidoreductases; EC 1.10.3.2), a multicopper oxidase widely distributed in plant and fungal species, can reduce oxygen directly to water through a four-electron transfer step, and this chemical reaction does not produce hydrogen peroxide (H_2_O_2_) [29]. Based on this, laccase has been utilized to fabricate a variety of biosensors, including biosensors for phenolic compounds [30]. Nafion, a linear perﬂuorosulfonate polymer possesses good cation-exchange properties, biocompatibility and film-forming properties and has been widely applied in the fields of fuel cells and sensors [31–32].

In the present work, we prepared ECNFs by carbonizing electrospun PAN nanofibers, and a novel catechol biosensor was fabricated through dropping a mixture solution made of ECNFs, laccase and Nafion on a processed glass-like-carbon electrode (GCE). Our results showed that the Laccase–Nafion–ECNFs sensor exhibited a noticeable eletrocatalytic ability towards catechol, and had a linear response range from 1 µM to 1310 µM with a detection limit of 0.63 µM, which all excelled most other laccase-based biosensors [33–36]. The biosensor was successfully applied in the detection of catechol in real water samples.

## Results and Discussion

### Morphology analysis

**Figure 1:**
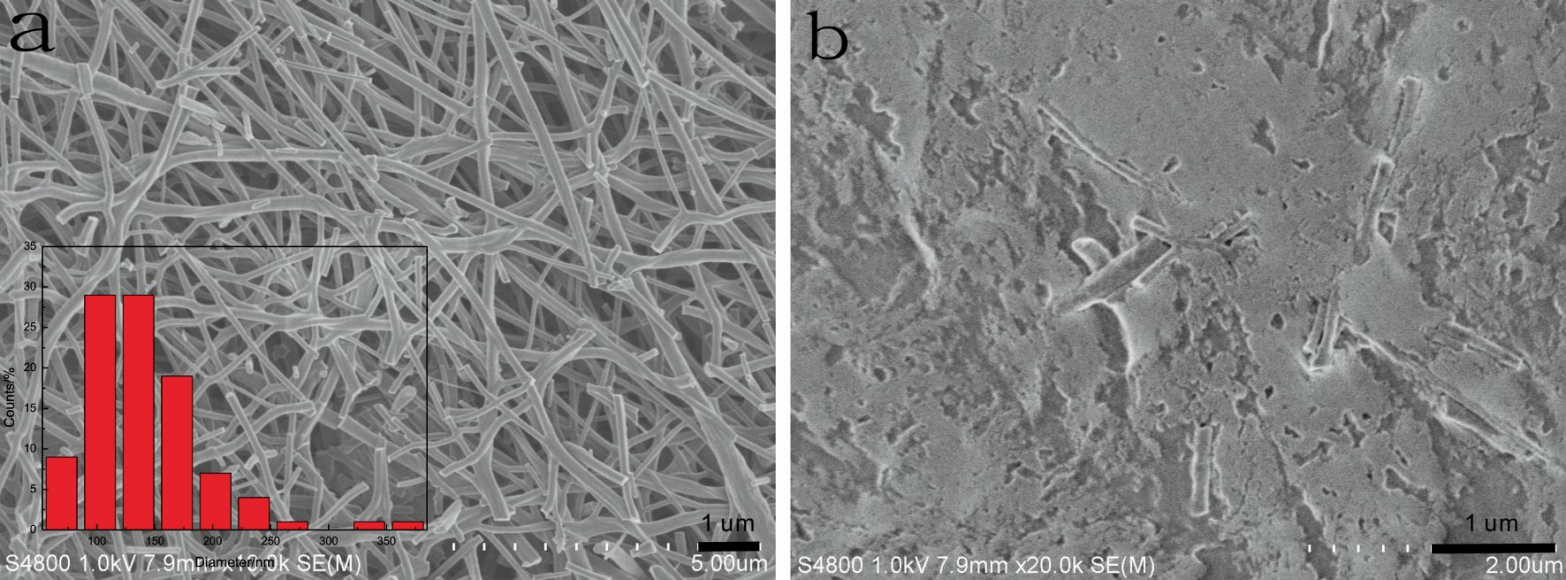
SEM images of the ECNFs (a) and the suface of laccase**–**Nafion–ECNFs/GCE (b). Insert: the diameter distribution diagram of the ECNFs.

The SEM images of ECNFs and laccase–Nafion–ECNFs/GCE are shown in Figure 1. As can be seen from Figure 1a, the randomly distributed ECNFs formed a fibrous web with an average fiber diameter of about 200 nm. The insert displays the diameter distribution of the ECNFs, which ranges from 50 to 380 nm and mainly focuses on 100 to 200 nm. Notably, many ECNFs were broken up into short fibers because the thermal treatment process enabled the fibers to become fragile. Figure 1b exhibits the surface morphology of the laccase–Nafion–ECNFs/GCE. It can be clearly seen that most of the short fibers were embedded into the laccase. Here, the short fibers may play a role of connecting the active center of laccase and the surface of GCE, which may be favorable for the electron transfer.

### Structure and component analysis

**Figure 2:**
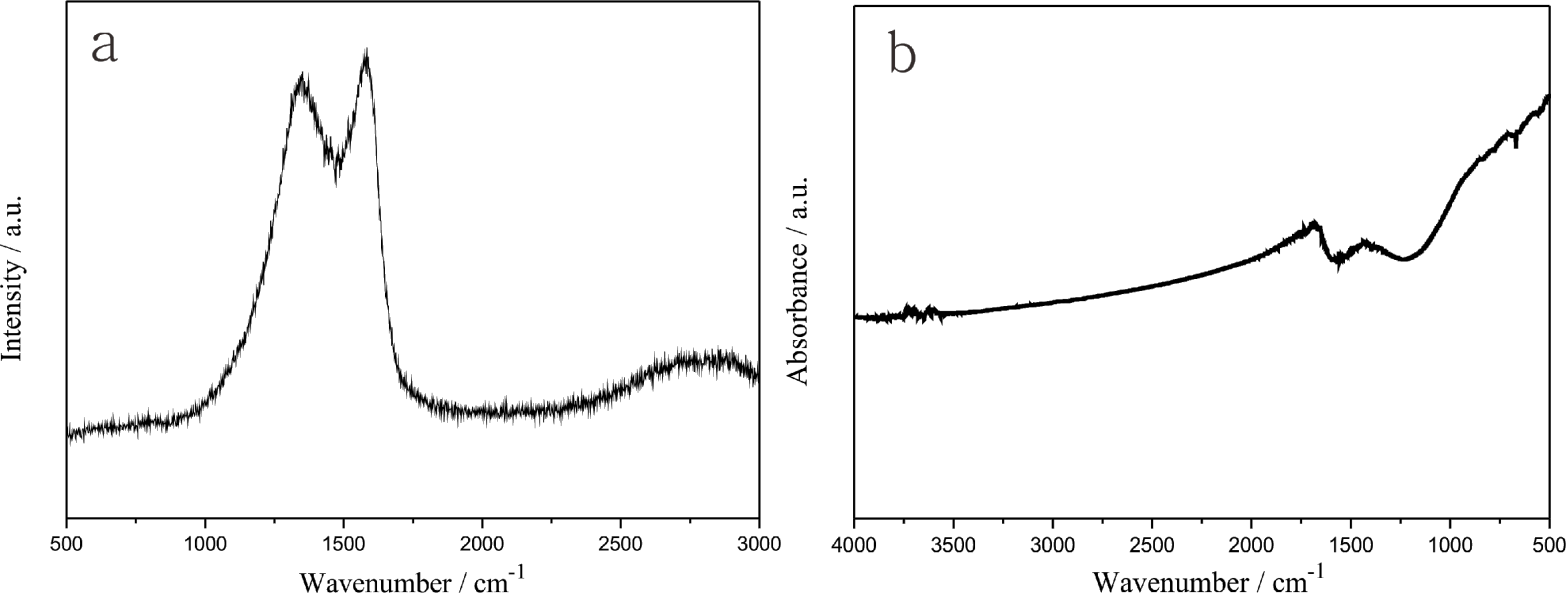
Raman spectrum (a) and FTIR spectrum (b) of the ECNFs.

The obtained ECNFs were, respectively, investigated by Raman and FTIR spectroscopy (Figure 2a and Figure 2b). As shown in Figure 2a, there are two characteristic peaks appearing at about 1330 and 1590 cm^−1^, which were related to the D-band and the G-band, respectively. The D-band was attributed to the defective carbon structure of the ECNFs, and the G-band could be ascribed to the in-plane carbon–carbon stretching vibrations of graphite layers [37]. This demonstrated that the ECNFs possessed polycrystalline structures and massive disordered and defected graphite layers. FTIR was employed to further study the functional groups on the surfaces of the ECNFs (Figure 2b). It is manifest that two distinct absorbance peaks, respectively, appeared at around 1710 and 1450 cm^−1^. And the two absorbance peaks were ascribed to the stretching vibration of the C=O bond of carboxyl [38] and the O–H bond [39]. This proved that there were numerous carboxyl groups on the surfaces of the ECNFs which were expected to improve the electrocatalytic properties and biocompatibility of the ECNFs [40].

**Figure 3:**
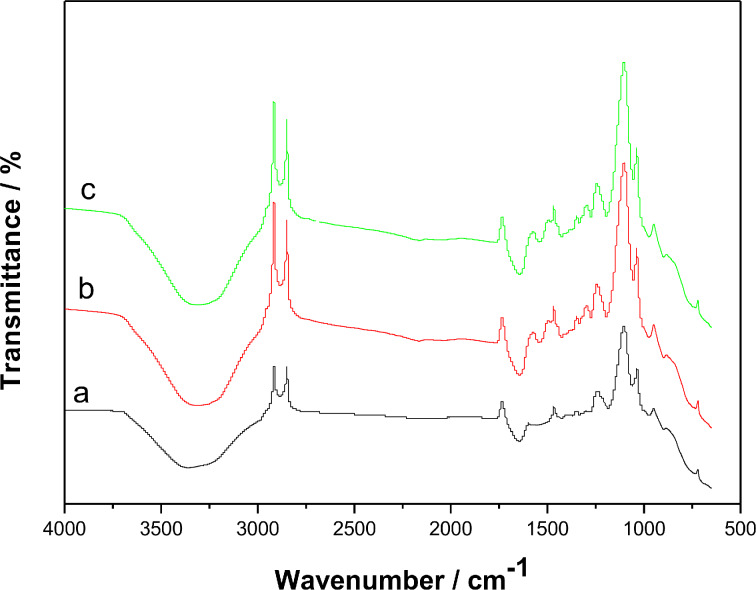
FTIR spectra of laccase (a), laccase**–**Nafion (b), and laccase**–**Nafion**–**ECNFs (c) thin films, respectively.

The three solutions, containing laccase, laccase–Nafion, and laccase–Nafion–ECNFs, respectively, were stored in refrigerator at 4 °C for two weeks and a certain amount of the three solutions was dropped onto a glass slide. Three thin films could be obtained after drying the solutions at room temperature. Figure 3 shows the FTIR spectra of the three dried thin films. The characteristic peak at approximately 1670 cm^−1^ could be attributed to the FTIR spectrum of the amide-I band of native laccase [41]. Similarly, some other bands, e.g., at 1066 and 1403 cm^−1^, which can also be ascribed to laccase, were observed in the three FTIR spectra of Figure 3a–c [41]. It can be clearly seen that the FTIR spectra of laccase–Nafion (Figure 3b) and laccase–Nafion–ECNFs (Figure 3c) solutions were similar with the one of pure laccase solution (Figure 3a), suggesting that laccase in the Nafion and the Nafion–ECNFs mixture both kept its activity and the ECNFs demonstrated their good biocompatibility with laccase. In addition, the laccase activity was also studied. The pristine laccase possessed an enzyme activity of 11.2 U/mg while this value decreased to 10.1 and 10.7 U/mg after the laccase was immobilized in the Nafion and Nafion–ECNFs, respectively. This also confirmed the good biocompatibility of Nafion and ECNFs and showed that the immobilization process had little influence on the activity of laccase.

### Direct electrochemistry and electrocatalysis of the laccase–Nafion–ECNFs/GCE

**Figure 4:**
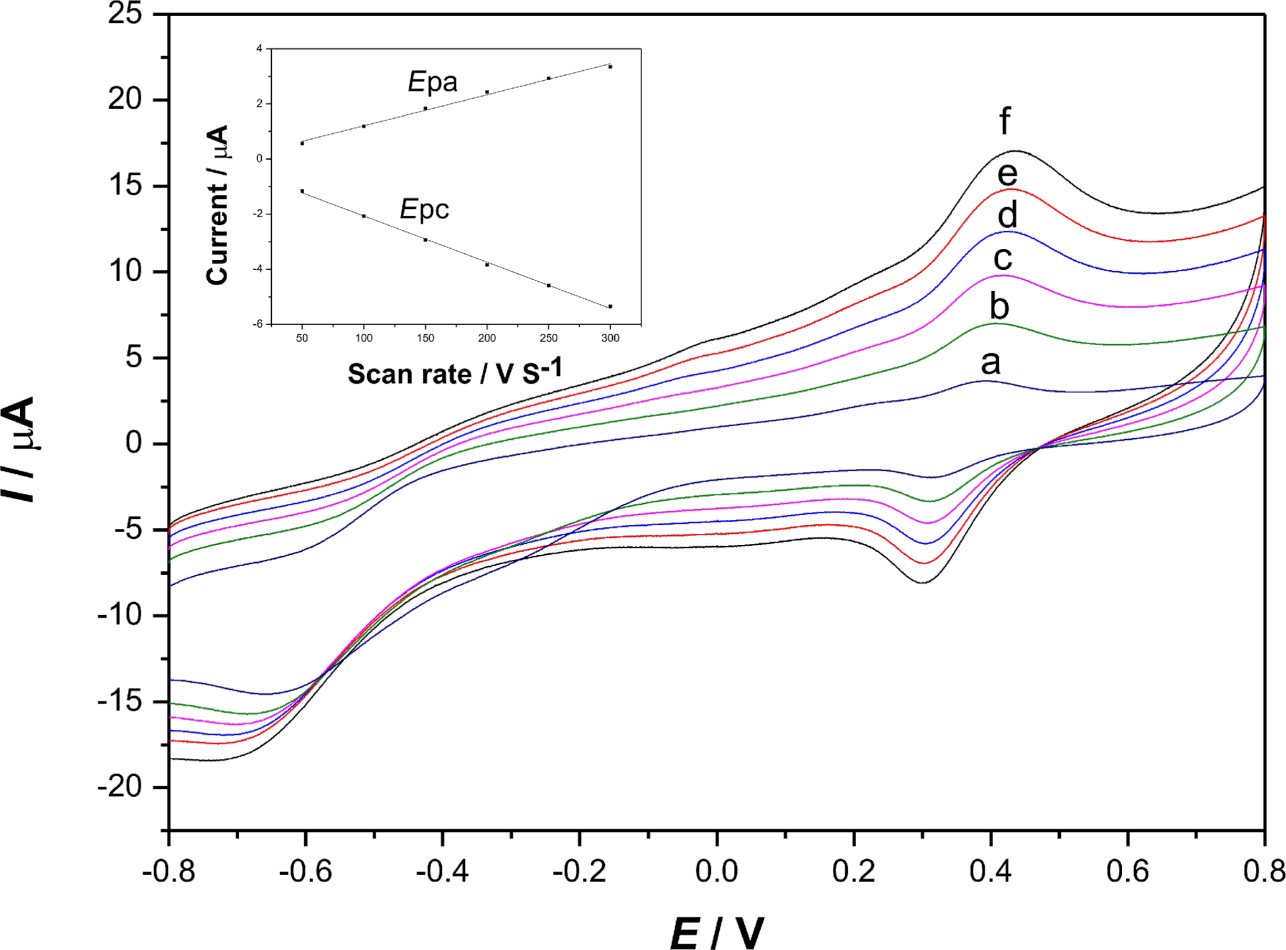
Cyclic voltammograms of the laccase**–**Nafion–ECNFs/GCE in acetate buffer (pH 4.0) with different scan rates (V·s^−1^): (a) 0.05; (b) 0.1; (c) 0.15; (d) 0.2; (e) 0.25; (f) 0.3. Inset: Calibration plot of anodic and cathodic peak currents vs scan rates.

Figure 4 presents the cyclic voltammograms of the laccase–Nafion–ECNFs/GCE in acetate buffer (pH 4.0) with scan rates from 0.05 to 0.3 V·s^−1^. It can be clearly seen that a pair of stable and well-defined quasi-reversible anodic and cathodic peaks occur, which can be attributed to the direct electron transfer between the laccase and the GCE. Besides, the anodic peak currents were larger than the peak cathodic currents, indicating a quasi-reversible electrochemical reaction process. Simultaneously, both of the currents increased with the rise of scan rates, the redox peak potentials shifted slightly with an increase in the distance between anodic peak and cathodic peak. As can be seen from the inset of Figure 4, the currents corresponding to redox peaks grew linearly with the scan rates from 0.05 to 0.3 V·s^−1^. This indicated that the electron transfer occurred easily between the laccase–Nafion–ECNFs composite and the surface of the GC electrode and that the electrochemical activity of the whole process is surface-controlled.

### Optimization of the enzyme electrodes

**Figure 5:**
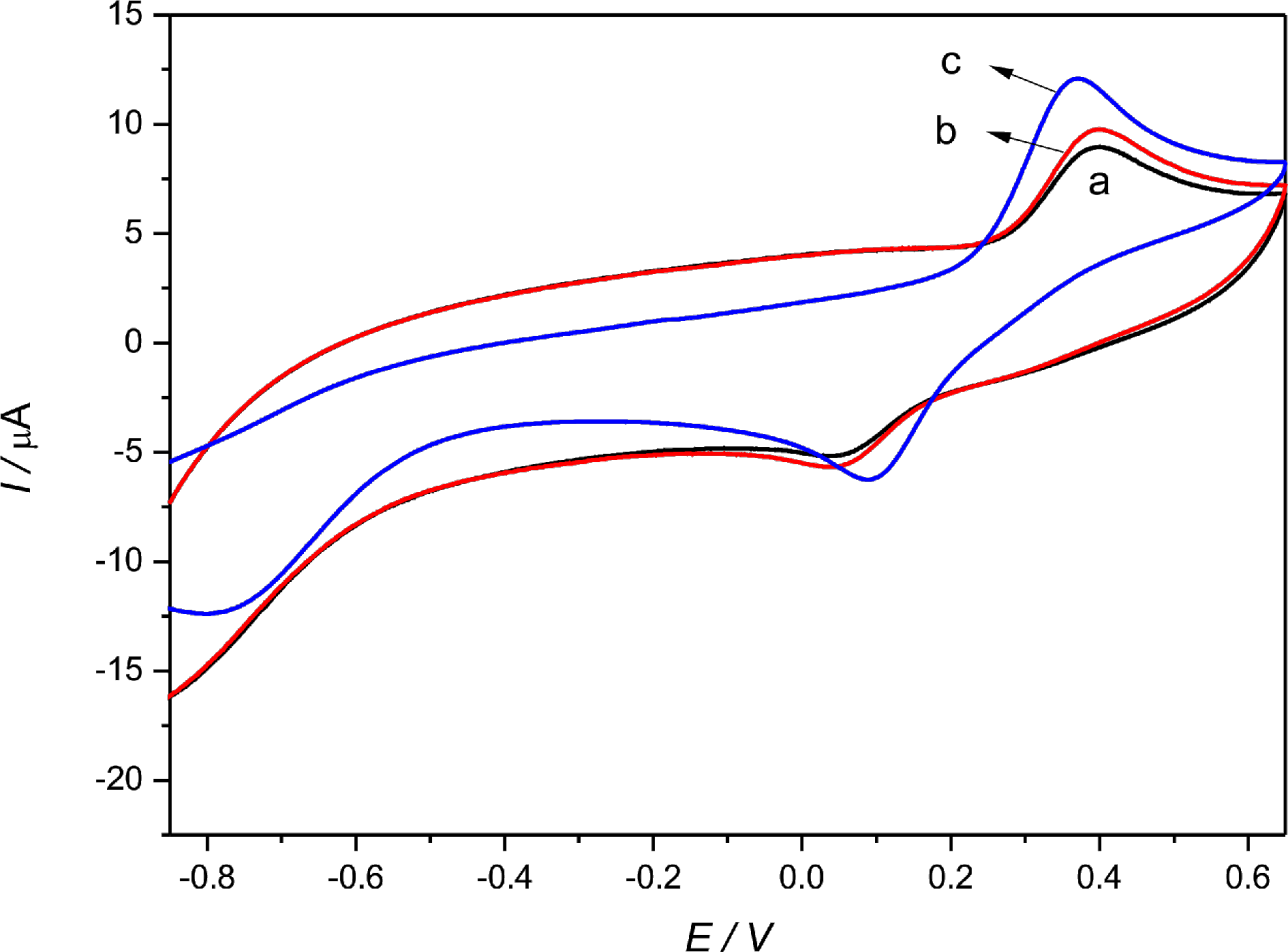
Cyclic voltammograms of of laccase**–**Nafion/GCE (a), laccase/GCE (b), laccase**–**Nafion–ECNFs/GCE (c) toward 300 µM catechol in 0.2 M acetate buffer (pH 4.0).

**Figure 6:**
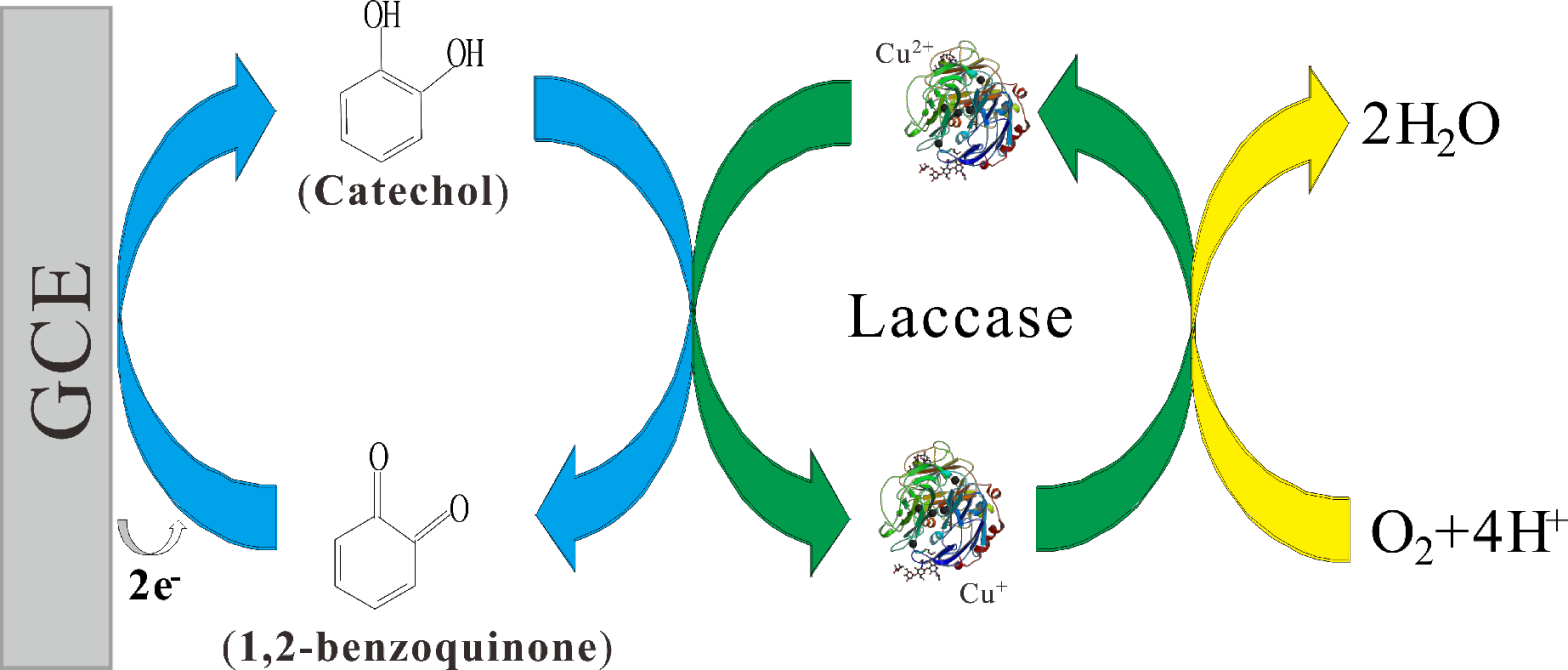
Schematic representation of laccase-catalyzed oxidation of catechol with its subsequent electrochemical reduction on the GCE.

Figure 5 shows the cyclic voltammograms of different enzyme electrodes toward 300 µM catechol in 0.2 M acetate buffer (pH 4.0). Compared with the peak current values of laccase/GCE (Figure 5b), those of the laccase–Nafion/GCE (Figure 5a) were smaller, which could be attributed to that Nafion impeded the transfer of electrons, to some extent. It is noticeable that the peak current values of the laccase–Nafion–ECNFs/GCE (Figure 5c) are larger than those of the laccase/GCE and the separation of peak potentials apparently decreased. This fully demonstrated that the ECNFs enhanced the conductivity of the composite and led to a faster electron transfer. The reaction mechanism is illustrated in Figure 6. First, the catechol on contact with the Lac was oxidized to 1,2-benzoquinone in the presence of molecular oxygen. Subsequently, the 1,2-benzoquinone was reduced electrochemically at the surface of the GCE.

**Figure 7:**
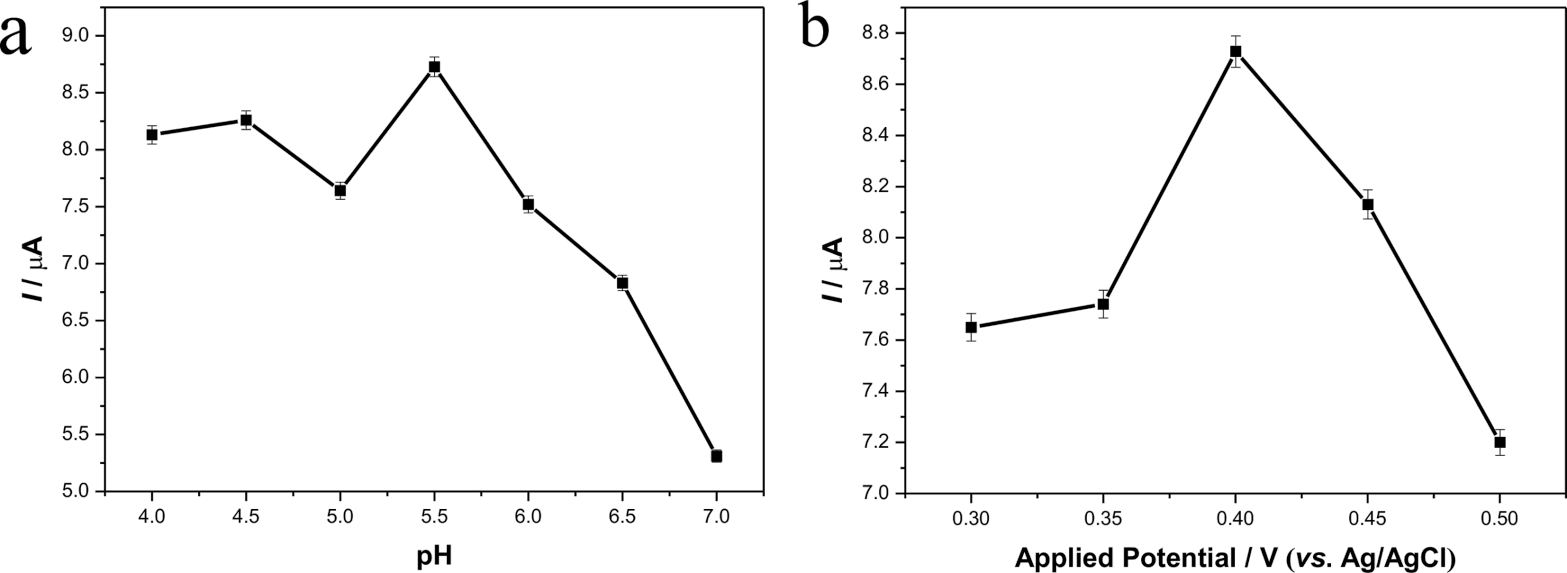
Influences of solution pH (at 0.4 V) (a) and applied potential (pH 5.5) (b) on the steady-state current response of 200 µM catechol in 0.2 M acetate buffer.

To acquire the optimal amperometric response, the effects of the pH value of the solution and of the applied potential on the current values were, respectively, investigated. As shown in Figure 7a, the current value reached the peak at pH 5.5, and then showed a dramatic decrease, which agreed with a previous report [34]. Figure 7b presents the influences of different applied potentials on the amperometric responses. It can be clearly seen that the maximum current value came at 0.4 V. So the applied potential was set at 0.4 V in the following experiments.

### Amperometric response of the biosensor

**Figure 8:**
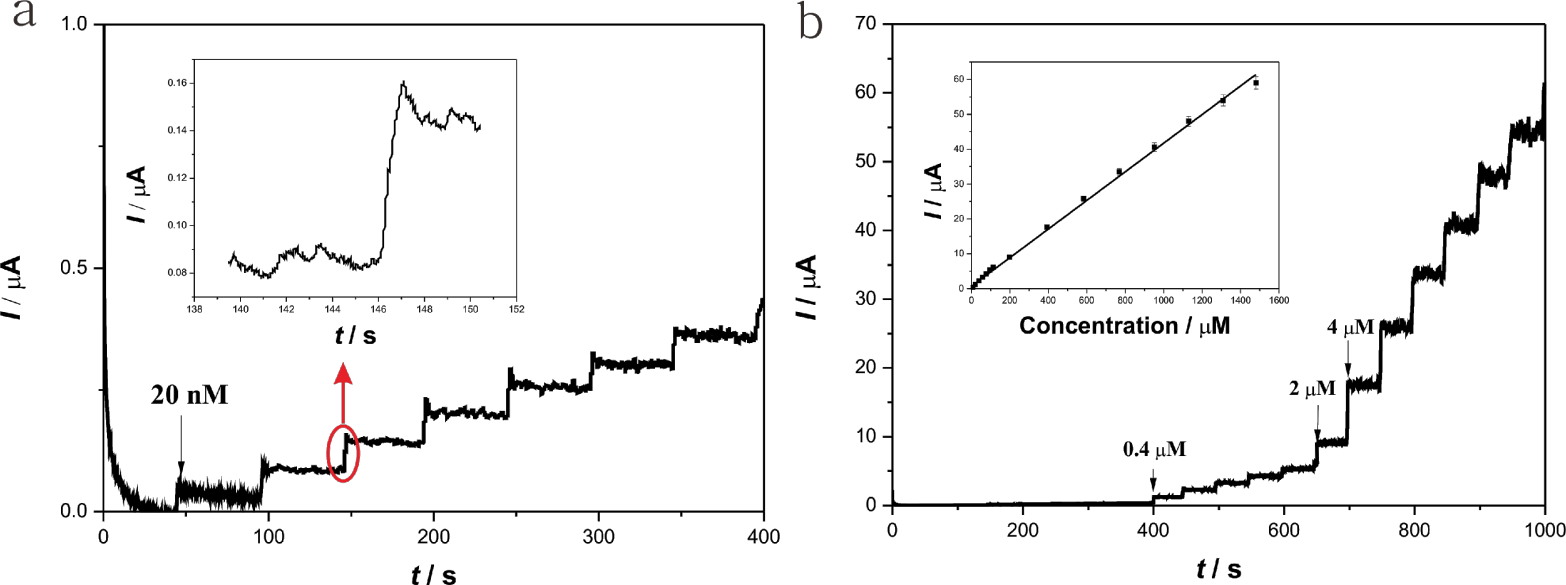
Typical steady-state current response of the laccase**–**Nafion–ECNFs/GCE on the successive addition of catechol solutions with different concentrations and volumes into 0.2 M acetate buffer (pH 5.5). Inset: A magnification of the third addition of catechol (a); The linear calibration curve of the current response on the catechol concentration (b).

The steady-state amperometric responses of the laccase**–**Nafion–ECNFs/GCE to different concentrations of catechol were determined by the successive addition of different volumes of 2 mM and 20 mM catechol into 20 mL acetate buffer (pH 5.5). It can be seen from Figure 8b that with the successive addition of catechol, the steady-state current values gradually increased. Figure 8a displays the magnified image of Figure 8b before 400 s, the first current step happened when adding 20 nM catechol into the acetate buffer. The insert in Figure 8a shows the rapid response of the biosensor toward catechol (attaining 95% of the value of the steady current within 2 s, which is shorter than in a previous report [35]). This sensitive response may be caused by the prompt diffusion of the analytes into the porous composite. The insert in Figure 8b shows the linear calibration curve of the current response on the catechol concentration. It can be seen that the response current increased with the increase in catechol concentration. The linear range was 1–1310 µM (R = 0.998, *n* = 19), which was much wider than for the biosensor based on CNTs and laccase [33]. And the sensitivity was 41 µA·mM^−1^, the detection limit was as low as 0.63 µM (S/N = 3). The apparent Michaelis–Menten constant (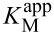) was estimated to be 50.64 µM according to the electrochemical version of the Lineweaver–Burk equation [42]. Table 1 compares several laccase-based biosensors. It can be seen that the laccase**–**Nafion–ECNFs/GCE exhibits a quite outstanding analytical performance and this new sensor could be useful in the detection of catechol.

**Table 1:** Performance comparison of different laccase modified electrodes.

electrode description	detection limit (µM)	linear range (µM)	sensitivity^a^ (µA·mM^−1^)	reference

laccase/CNTs–CS/GCE	0.66	1.2–30	–	[33]
Lac/AP-rGOs/Chit/GCE	7	15–700	15.79	[34]
MB-MCM-41/PVA/lac	0.331	4–87.98	–	[35]
Cu-OMC/Lac/CS/Au	0.67	0.67–13.8	104	[36]
laccase–Nafion–ECNFs/GCE	0.63	1–1310	41	this work

^a^The dash indicates no reported value.

### Interferences and biosensor stability

**Figure 9:**
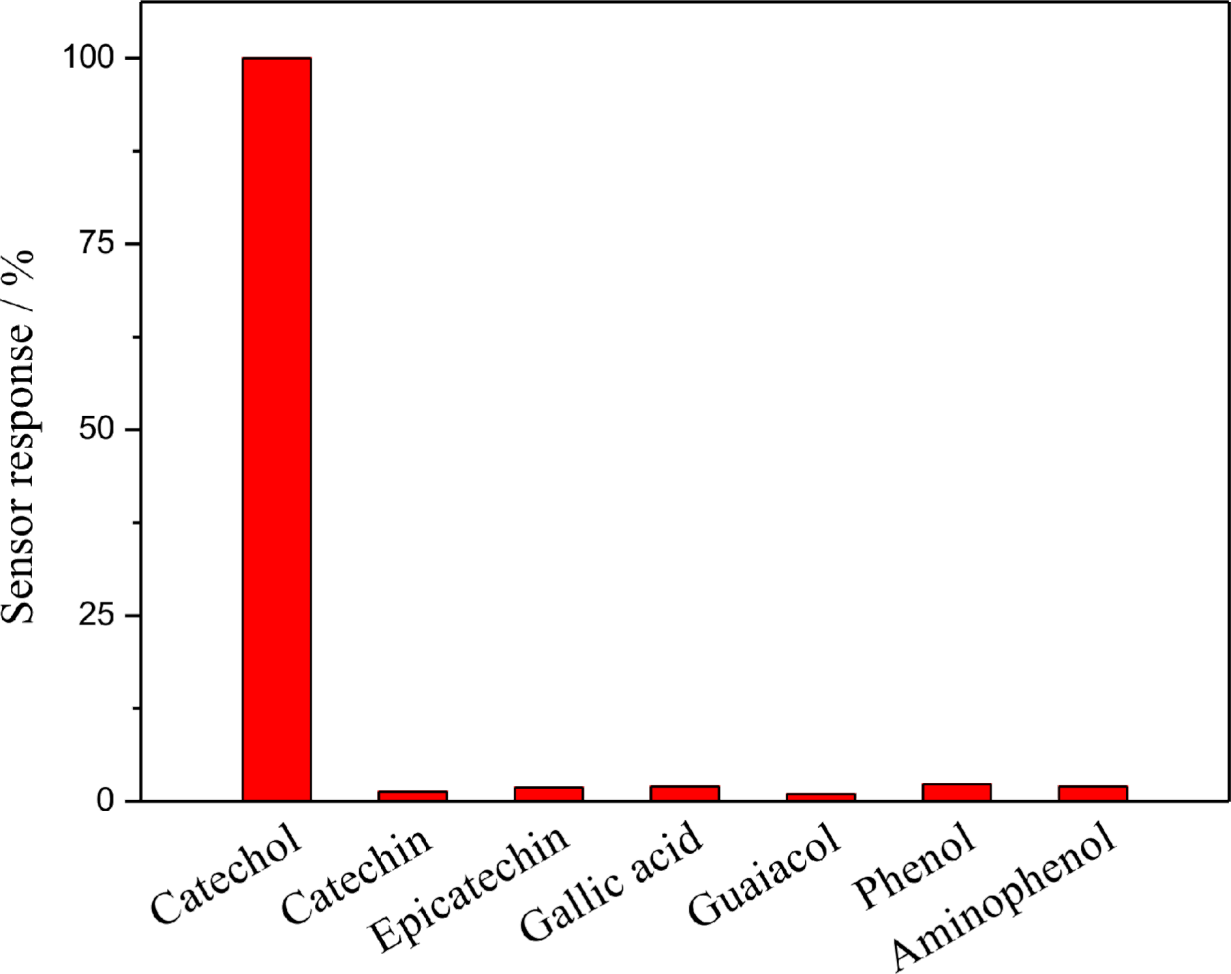
Relative responses of the laccase**–**Nafion–ECNFs/GCE for different phenolic compounds (catechol, catechin, epicatechin, gallic acid, guaiacol, phenol and aminophenol; 100 µM in 0.2 M acetate buffer (pH 5.5), respectively).

Catechol and some other phenolic compounds, including catechin, epicatechin, gallic acid, guaiacol, phenol and aminophenol, were used to determinate the selectivity of the biosensor (Figure 9). The biosensor showed excellent selectivity for catechol (set to 100%) and exhibited almost no response to other phenolic compounds.

**Figure 10:**
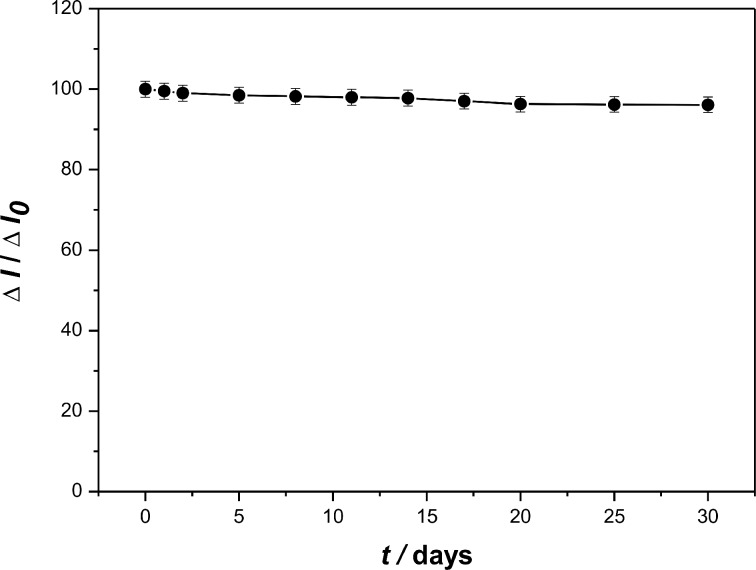
Storage stability of the laccase**–**Nafion–ECNFs/GCE in 0.2 M acetate buffer (pH 4.0) at 4 °C.

The biosensor showed good repeatability, reproducibility and stability. The biosensor was used to measure successively for 10 times in a certain concentration of catechol solution, and the relative standard deviation (RSD) of the response current value was within 2.0%, which indicating the biosensor possessed good repeatability. Besides, we prepared five biosensors under the same conditions, and the RSD of the response current values of five modified electrodes was 3.5%, which indicates that the biosensor had acceptable reproducibility. Figure 10 shows the storage stability of the laccase**–**Nafion–ECNFs/GCE in 0.2 M air-saturated acetate buffer (pH 4.0) at 4 °C. It is manifest that over a storage period of one month, the current response only decreases slightly. Even after 30 days, the current response retained 96.3% of the initial value, which indicated that the laccase preserved its activity well in the mixture of Nafion and ECNFs and that the biosensor possessed good stability.

### Application to water samples

To demonstrate the practical application of the catechol sensor, the response of the sensor in water samples was investigated. As shown in Table 2, based on the equation of calibration curve, *I* = 0.041*c* + 0.668 (*I* in µA, *c* in µM), the corresponding amount of catechol could be calculated. The recoveries looked satisfactory, confirming that the biosensor can realize its practical application in detecting phenols in real samples.

**Table 2:** Recovery experiment of detection of catechol in real water samples.

sample	*c*_added_ (µM)	*c*_found_ (µM)	recovery (%)	RSD (%)

tap water	100	101.7	101.7	1.6
		99.2	99.2	
		99.7	99.7	
		98.6	98.6	
		97.3	97.3	
Taihu Lake water	100	103.4	103.4	3.5
		102.1	102.1	
		96.1	96.1	
		97.2	97.2	
		96.5	96.5	

## Conclusion

Carbon nanofibers with excellent electrochemical properties and biocompatibility were fabricated by electrospinning and high temperature carbonization techniques. And the ECNFs were employed to design a novel laccase-based biosensor, which displayed outstanding sensitivity to catechol with a wide linear range, a low detection limit and a fast response. Furthermore, the biosensor also displayed good repeatability, reproducibility and stability, and was successfully applied in detecting catechol in real samples.

## Experimental

### Materials

Laccase, Nafion and 2,2’-azinobis(3-ethylbenzothiazole-6-sulfonic acid) (ABTS) were purchased from Sigma-Aldrich. Other chemical reagents were obtained from Sinopharm Group Chemical Reagent Co., Ltd. (Shanghai, China). All reagents were analytical grade and used without further purification. All aqueous solutions were prepared with Milli-Q purified water (>18.0 MΩ·cm). The acetate buffer system (containing 0.2 M HAC-NaAC) was selected as buffer solution.

### Apparatus

A Hitachi S-4800 field-emission scanning electron microscope (FE-SEM) was used to examine the surface morphologies of the ECNFs and the laccase–Nafion–ECNFs/GCE. The Raman spectrum analysis was carried out at room temperature using a 3D Nanometer Scale Raman PL Microspectrometer (Tokyo Instruments, Inc., with a 785 nm He–Ne laser). Fourier transform infrared (FTIR) spectra were recorded in the range of 500–4000 cm^−1^ on a Nicolet iS10 FTIR spectrometer (Thermo Fisher Scientific). Electrochemical experiments were carried out at room temperature by using a CHI 660D electrochemical workstation (CH Instruments, Inc., Austin, USA). A UV spectrophotometer (UNNICO Instruments Co., Ltd., Shanghai) was used to calculate the activity of laccase.

### Preparation of ECNFs

The ECNFs were prepared by the following steps. Firstly, the electrospinning solution was prepared by dissolving 10 wt % polyacrylonitrile (PAN) powders in DMF with magnetic stirring for 8 h. Secondly, the prepared solution was added into a syringe for electrospinning. The experimental parameters were set to a voltage of 15 kV, a working distance of 15 cm, and a flow rate of 0.5 mL/h respectively. Lastly, a high temperature furnace was employed to stabilize and carbonize the PAN nanofibers. The whole process was conducted in N_2_ atmosphere and could be divided into two phases: (1) Heating up to 300 °C at a rate of 2 °C·min^−1^ and keeping this temperature for 2 h. This process was for stabilizing the shape of nanofibers. (2) Heating up to 1000 °C at a rate of 5 °C·min^−1^ to carbonize the nanofibers, keeping the highest temperature for 2 h, and then cooling down to room temperature.

### Preparation of the modified electrodes

Considering the current response and the stability of modified electrode, in control experiments, the concentrations and mass ratio of Naﬁon, ECNFs and laccase were optimized. Ultimately, the biosensor was fabricated by using a mixture containing 1.5 wt % Naﬁon, 0.4 mg·mL^−1^ ECNFs and 3 mg·mL^−1^ laccase.

A typical procedure for the preparation of the laccase–Nafion–ECNFs/GCE is as follows: First, with the help of ultrasonication and stirring, 4 mg ECNFs is added into 10 mL acetate buffer (pH 4.0) to obtain ECNFs suspension. Next, a mixture containing a certain volume of Naﬁon (5 wt %), ECNFs suspension and the appropriate mass of laccase was kept stirring for 1 h. Finally, the laccase–Nafion–ECNFs/GCE was prepared by dropping 10 µL of the mixture onto the surface of a freshly polished glass carbon electrode. The glass carbon electrode was processed as follows: Firstly, it was polished with alumina. Following that, it was rinsed by water and ultrasonicated in ethanol and water. Finally, it was dried under a nitrogen atmosphere. The dried laccase–Nafion–ECNFs/GCE was kept in storage at 4 °C.

Meanwhile, laccase**–**Nafion/GCE and laccase/GCE were prepared to compare with the laccase–Nafion–ECNFs/GCE. The laccase/GCE was prepared by using a solution containing 3 mg·mL^−1^ laccase and the laccase-Nafion/GCE was prepared by using a solution containing 1.5 wt % Nafion and 3 mg·mL^−1^ laccase. Herein, the mass of laccase in different electrodes should be kept equal. In addition, all the electrodes were dipped into acetate buffer (pH 4.0) for 30 min to remove the unstable compounds before electrochemical measurements.

### Determination of the activity of free and immobilized laccase

To investigate the effect of the immobilization process on the laccase activity we, respectively, studied the free and immobilized laccase activity according to the reported method [43]. The activity of laccase was determined by the UV spectrophotometer at 420 nm using ABTS as the substrate. One unit of laccase activity was deﬁned as the amount of laccase to catalyse 1 µM of ABTS per minute.

### Preparation of water samples

To start with, a microporous membrane was used to filter the prepared water samples (tap water from our lab and water from Taihu Lake). Next, the filtered water samples were added into 0.2 M (pH 5.5) acetate buffer to dilute them (double dilution). Afterward, the diluted water samples were added into 20 mL of 0.2 M air-saturated acetate buffer (pH 5.5). Finally, an amperometric detection (repeated five times) based on the laccase**–**Nafion–ECNFs/GCE at 0.4 V was conducted after adding 100 µM catechol into the solutions.
